# Placement of LC-II and trans-sacral screws using a robotic arm in a simulated bone model in the supine position – a feasibility study

**DOI:** 10.1186/s40634-022-00476-w

**Published:** 2022-04-27

**Authors:** Jon B. Carlson, Jiyao Zou, Brandi Hartley

**Affiliations:** Department of Orthopaedic Surgery, University of Louisville, 550 S. Jackson St 1st Floor ACB, Louisville, KY 40292 USA

**Keywords:** Robot, Robotic, Fracture, Pelvis, Acetabulum, Pelvic

## Abstract

**Purpose:**

The use of a robotic arm has been well-described in the literature for the placement of pedicle screws in spine surgery as well as implants for sacroiliac joint fusion. There are no reports describing the use of a robotic arm to place screws in osseous fixation pathways (OFPs) employed in the treatment of pelvic ring and acetabular fractures outside of a single center in China. Using a Sawbones model, the authors describe a technique for using a robotic arm widely available in Europe and the Americas for placement of 6.5 mm cannulated screws into two OFPs commonly used in the treatment of pelvic and acetabular fractures.

**Methods:**

Using the Mazor X Stealth Edition (MSXE) robot from Medtronic, the authors were able to place a pin into the pelvis onto which the robot was docked. The authors were then able to designate the area of interest using navigated instruments, and in combination with the MSXE “scan and plan” marker, obtain cross-sectional imaging using the O-Arm and successfully register the MSXE robot. We then used the provided software to plan trajectories for the lateral compression type 2 (LC-II) screw pathway as well as a pathway for a trans-ilio-trans-sacral screw. We describe in detail the steps for setup, planning and placement of 6.5 mm cannulated screws using the MSXE robotic arm into these two OFPs.

**Results:**

Visual inspection and plain x-rays demonstrated successful placement of the screws into the two planned OFPs. No breach of cortical bone was seen on either visual inspection of the model or demonstrated on post-procedure x-rays.

**Conclusion:**

It is possible to use the Mazor X Stealth Edition robot to place screws into the LC-II and trans-ilio-transsacral screw pathways in a Sawbones model. This is only a feasibility study, and should in no way be taken to suggest that clinical application of this technique should be attempted.

## Background

Placement of 6.5 mm cannulated screws for the fixation of pelvic and acetabular fractures is standard practice in orthopaedic trauma surgery. Contemporary techniques for placement of screws in pelvic and acetabular surgery employ extensive use of fluoroscopy exposing the surgeon, residents, patients and operating room staff to high levels of ionizing radiation [1]. Placement of these screws is technically challenging with a steep learning curve for newly-minted trauma surgeons. A misplaced screw crossing the S1 or S2 neural foramen or injuring neurovascular structures anterior to the sacrum at this level can lead to severe injury or death due to damage to neurovascular structures. As in the spine – if not more so – the 3D anatomy of the pelvis, acetabulum and sacrum are extremely complicated. Correlation of the 2D images provided by fluoroscopy intraoperatively to the 3D anatomy of the pelvis can take years of practice. Additionally, it is sometimes impossible to obtain adequate fluoroscopic imaging in the OR due to various factors such as overlying bowel gas and patient body habitus. The inability to obtain adequate imaging is an absolute contraindication to placement of the screws due to the possibility of devastating injury with screw malposition [2].

The fluoroscopic views necessary for the placement of various screws for fixation of pelvic and acetabular fractures have been described [3]. The use of various imaging guidance techniques for placement of these implants including 2D and 3D fluoroscopy and CT navigation have also been described. While not reaching statistical significance, a meta-analysis in 2013 demonstrated the lowest rate of mal-positioned screws with the use of CT navigation [4]. CT navigation is also routinely used for placement of pedicle screws during spinal surgery with studies demonstrating relative safety and accuracy of CT navigation for placement of these implants [5, 6]. When using CT navigation, the instruments are positioned using a freehand technique with the surgeon correlating positioning of the instruments in space with a virtual display representing the projected trajectories of the instruments or screws. The use of a freehand technique introduces the potential for inaccuracies between planned and actual trajectories given that the instruments are used without a fixed guidance system such as a targeting jig for placement of interlocking screws for a femur or tibia nail. Multiple manufacturers have developed a robotic arm to aid in the placement of pedicle screws in the spine. This arm can be positioned according to pre-planned screw trajectories and functions as a real-time custom jig for the placement of cannulas, instruments and implants for spinal surgery in a similar way as a targeting jig allows a surgeon to place interlocking screws into a femoral or tibial nail. The key difference is that the robotic arm can be positioned in space at the surgeon’s discretion in any way relative to the bony anatomy given the limits of the articulations of the arm and the soft tissues. The use of the robotic arm for placement of pedicle screws has also been shown to be relatively accurate vs non-robotic CT navigation, fluoroscopically-aided or purely freehand techniques [7–11]. The use of a robotic arm to place percutaneous screws for pelvic ring injuries has been described by Liu, et al. using the TiRobot (TINAVI Medical Technologies, Beijing, China) [12]. However, there are no reports describing the use of robotic arms that are widely available in Australia, Europe or the Americas to place screws into OFPs commonly used in the fixation of pelvic and acetabular fractures. The purpose of our feasibility study is to explore possible applications of a robotic arm for placement of instruments and implants for screws commonly placed for pelvic and acetabular surgery. The screw trajectories evaluated in this feasibility study used the trans-sacral osseus fixation pathway at the level of the first sacral segment and LC-II osseus fixation pathway in a simulated bone model (Sawbones, Vashon Island, WA).

## Methods

### Room and robot setup

**Fig. 1:**
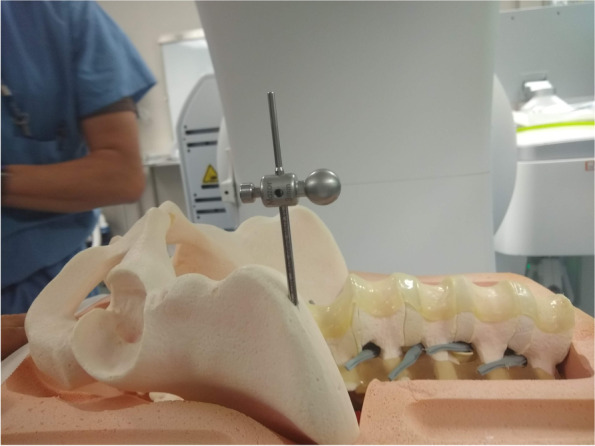
Lateral view of pin placement for attachment of the robot to the model

The simulated bone model was placed supine on a radiolucent table and taped in place (Fig. 1). For the purposes of this feasibility study, the simulated soft tissue envelope was removed from the anterior aspect of the model. We utilized the Mazor X Stealth Edition (MXSE) robotic arm (Minneapolis, Minnesota). The surgical planning, operating room setup, robotic arm mounting and registration of the robot has been described, and except as necessary for this novel use of the robot, was followed [13]. Pre-procedure cross-sectional imaging was obtained using the O-Arm with a “scan and plan” marker to simulate an O-Arm spin in the operating room just prior to the procedure. The base of the robotic arm was initially positioned toward the head, but that lead to difficulty achieving the necessary position of the arm for placement of the screws. Subsequent setup positioned the base of the robotic arm toward the feet which allowed the arm to be positioned as needed for placement of the screws.

### Surgical technique - preparation

A pin must be placed into the pelvis to allow for attachment of the robot to the pelvis. There are several options for pin placement, all of which are off label, as is the entire technique we describe. Regardless of the chosen starting point, it is important to note that the pin must be placed perpendicular to the floor and parallel with the walls of the OR (“straight up and down”) to allow for proper attachment of the robot. Options for the start point include: The inferior aspect of the anterior inferior iliac spine (AIIS), anticipating an LC-II osseus fixation pathway (OFP) screw more superiorly; the superior aspect of the AIIS, anticipating an LC-II screw more inferiorly; the anterior superior iliac spine (ASIS); the iliac crest superior and posterior to the ASIS. For the purposes of this feasibility study, the pin was placed in the anterior to posterior direction at the iliac crest anteriorly and just superior to the iliac spine.

### Surgical technique - registration

For the purposes of this feasibility study, we used a “scan and plan” technique, meaning that O-arm imaging and robot registration was performed after the model was secured to the operating table immediately prior to the procedure rather than planned from a pre-procedure CT. With this robot, the markers used for registration of the robot are different with “scan and plan” using an O-Arm spin after positioning the model on the OR table vs obtaining an actual CT with a standardized protocol in advance of the simulated procedure. Registration was carried out according to manufacturer’s directions under the supervision of the robot technician. The pin was placed as described above, and the robot was docked to the model. The model was covered by a blue-colored operative gown, and a visual scan was done by the robotic arm to prevent collision with the simulated soft tissues. The robot was attached to a pin placed into the pelvis (Fig. 1). The navigation guide for the arm was placed and registered. Next, the “turkey foot” pointer was brought into the navigation field and held over the central area of interest for the O-Arm scan. Next, the robotic arm was brought into position as indicated by the central area of interest in preparation for the O-Arm scan. The star-shaped “scan and plan” acrylic guide was then attached to the arm. These steps completed the necessary preparation for the O-Arm spin. The O-Arm was then brought in and positioned over the area of interest. 2D Fluoroscopy images with the O-Arm were obtained in the AP and lateral planes to include the four metallic spherical markers contained in the acrylic guide as well as the simulated bony anatomy of interest. We used a 40 cm window for the O-.

Arm. At the time of this writing, only high-dose scanning was available as a setting when using a 40 cm (vs 20 cm) window for the O-Arm. Such a high setting with use of a sawbones model led to overexposure of the scan, but we were able to adjust the contrast and brightness settings of the MXSE software to visualize the simulated bony anatomy to a degree needed to plan, execute and evaluate screw placement. Of note, the four metallic spherical markers must be contained within the 2D window on all O-Arm fluoroscopic views for the purposes of registration. Even though the 40 cm window extends beyond the fluoroscopic views in 2D, registration was not possible if they are not contained within the fluoroscopic views even if they are contained within the 40 cm scan. Once the spin was complete, the images were transferred to the MXSE robot, and a successful registration of the robot was achieved.

It is important to note that on the O-Arm settings, the model (patient) was indicated to be in the SUPINE position.

It is equally important to note that for the MXSE robot software, the model (patient) was indicated to be in the PRONE position. At the time of this writing, no supine option is available with the MXSE software.

This software limitation can be overcome as discussed in the next section.

### Surgical technique – planning

Once the O-Arm spin has been acquired and transferred to the MXSE robot we began to plan the screws. One of the first steps for the planning for spine surgery involves the robot technician outlining each spinal segment. We found that it is best to define the entire scan as a single “S1” segment that includes all bony anatomy of interest obtained from the O-Arm spin. This selection allowed us to utilize all the scanned anatomy for planning purposes.

A key concept of the current feasibility technique involves “scan and plan” once the patient (model) has been transferred to the table. With unstable sacral, sacroiliac, pelvic ring and/or acetabular fractures, any positioning and reduction techniques would need to be employed and maintained during and after the O-Arm spin to avoid mal-positioning of instruments or implants due to shifting of the bony anatomy following acquisition of the cross-sectional imaging. It is currently unknown whether a pre-operative CT scan could be used to plan screw trajectories prior to transfer to the OR table.

**Fig. 2:**
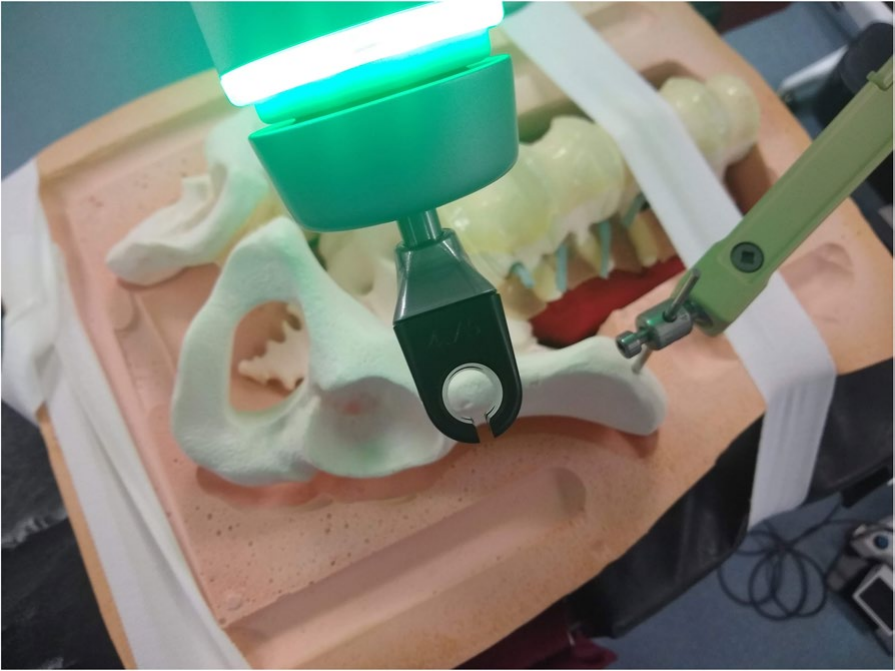
View down robotic arm in preparation for LC-II screw

The next and one of the most critical steps is that, due to software limitations at the time of this writing, it is necessary to “trick the robot.” Part of the robot technician’s job is to tell the robotic software which direction of the scan is anterior, and which direction is posterior. One of the key findings of our feasibility study is that if the model is in the supine position, the robotic arm will not work correctly if the robotic planning software designates true anterior and true posterior. The work-around for lack of “supine” as an option for “patient position” is simply to tell the robot that anterior is posterior, and posterior is anterior. The robot technician can setup the case just as she usually would. Then, on the axial views, switch anterior to posterior by rotating that axis 180 degrees. Of note, this also leads to the right side being marked as the left side by the robotic software and vise a versa. There is no currently other available software work-around available as far as the authors are aware. Simple visual inspection when moving the robotic arm to a planned start point for a screw will immediately reveal if right and left sides have been inadvertently interchanged during planning, and the plan can be easily revised to correct any such mistakes in the plan. Once the planned LC-II trajectory was entered into the robot, the robotic arm was sent to the planned trajectory. Direct visualization down the canula demonstrated proper positioning of the robotic arm for placement of the instruments and screw for this osseus fixation pathway with the anterior inferior iliac spine positioned exactly in the center of the robotic canula (Fig. 2).

### Surgical technique – instrumentation and placement of screws

**Fig. 3:**
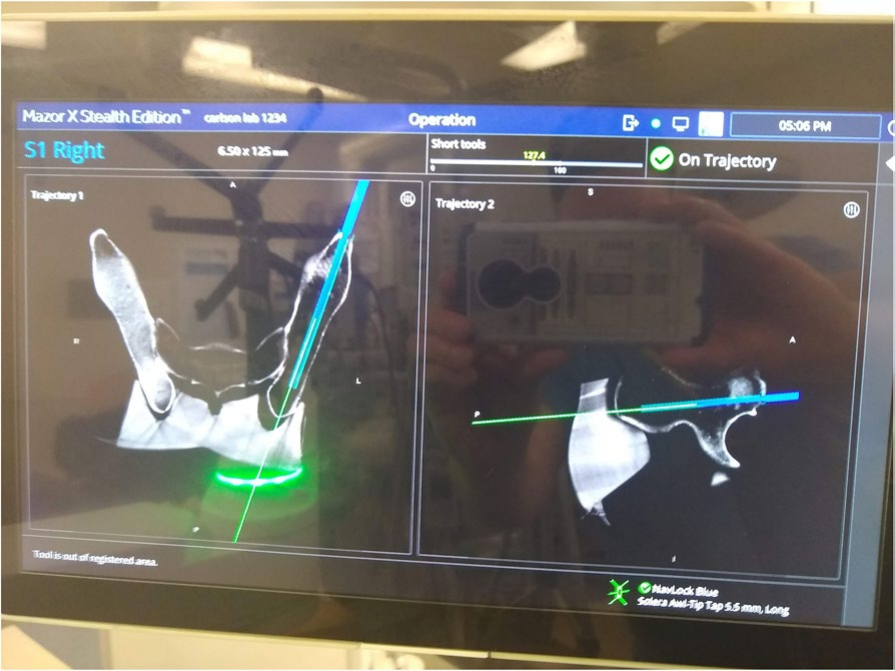
Burr ready to prepare the starting point for the LC-II screw with virtual trajectory indicated

**Fig. 4:**
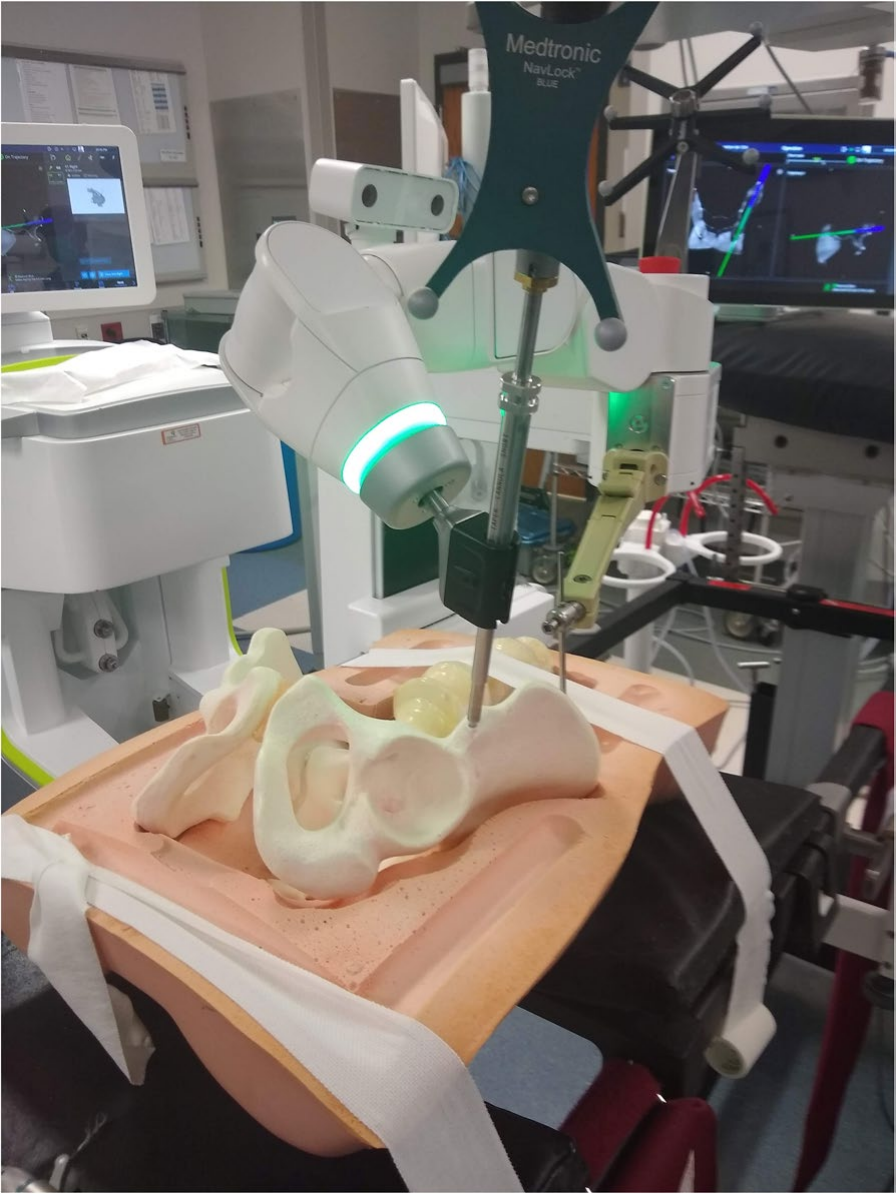
Tap diameter 5.5 mm seated to the shoulder to prepare initial screw trajectory

**Fig. 5:**
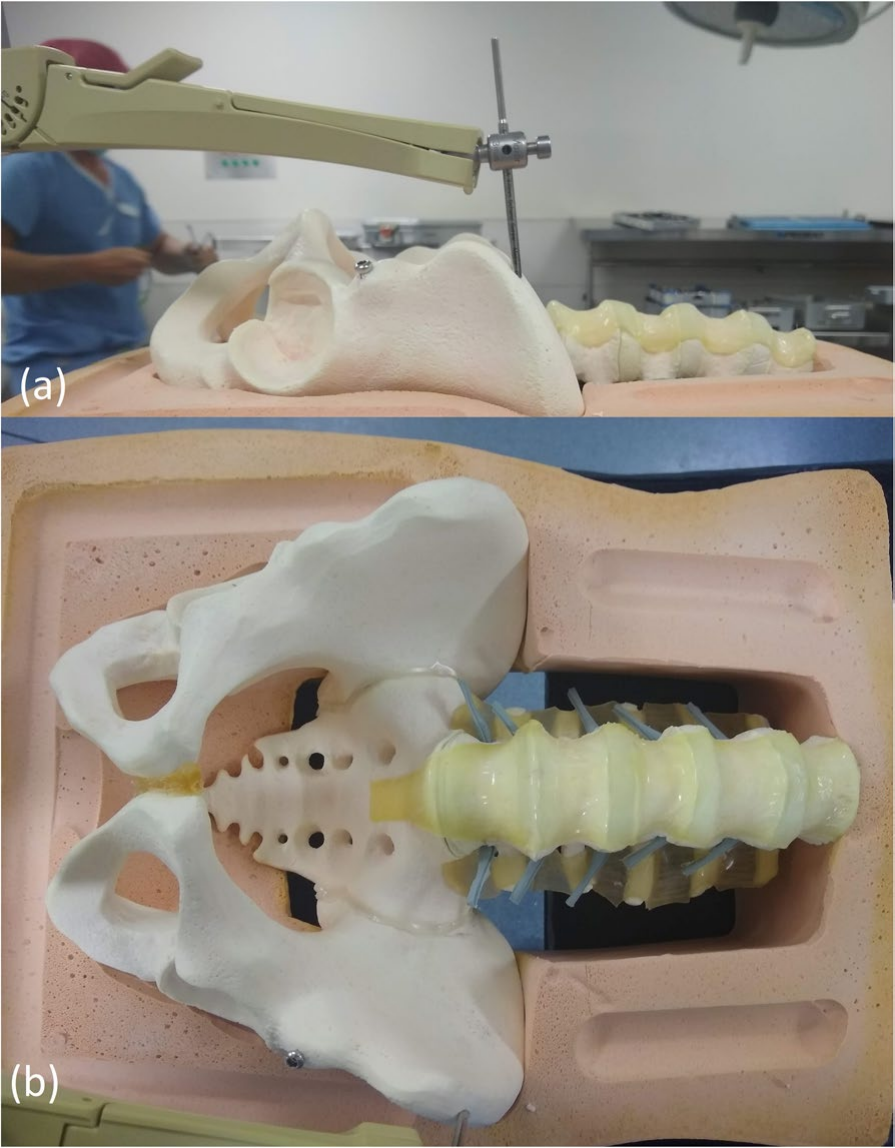
Lateral (**a**) and AP (**b**) pictures of the model with the LC-II screw in place

Once the robotic arm has been sent to the trajectory for the first screw, cannulas are placed to allow an opening burr to open the outer cortex of the bone (Fig. 3). Use of the burr serves to minimize deflection, skiving of the awl-tipped tap, and maximizes accuracy of the starting point. The implants chosen for this feasibility study were 6.5 mm cannulated screws from Synthes (West Chester, Pennsylvania) with an inner diameter of 4.8 mm. Due to concern of the possibility of the guidewire deviating from the planned trajectory due to its flexibility, we used a 5.5 mm awl-tipped tap from the robot manufacturer that is natively navigated to establish the initial 5–6 cm of the planned trajectory for the screws. Fig. 4 demonstrates seating of the tap to the limit of the shoulder of the tap. The tap was removed and triple sleeve nested cannulas for the insertion of a guidewire for a pedicle screw were placed, but with the inner-most sleeve removed to allow for placement of the 2.8 mm drill tip wire for the 6.5 mm screw. Due to concerns with wire migration the 2.8 mm wire was not driven further into the model with a wire driver. With the wire in place, the limits of the osseus fixation pathway were felt in all directions using the guide wire as one might use a pedicle feeler probe. Bone was felt circumferentially. A solid endpoint within the bone was noted. The cannulas for the wire were removed and the screw was placed over the wire and advanced by hand. Lengths of the screws were determined by available implants. With greater implant availability, screw lengths could be planned with the robotic planning software. Figure 5 demonstrates placement of the screw without any cortical violation according to the planned trajectory for the implant.

**Fig. 6:**
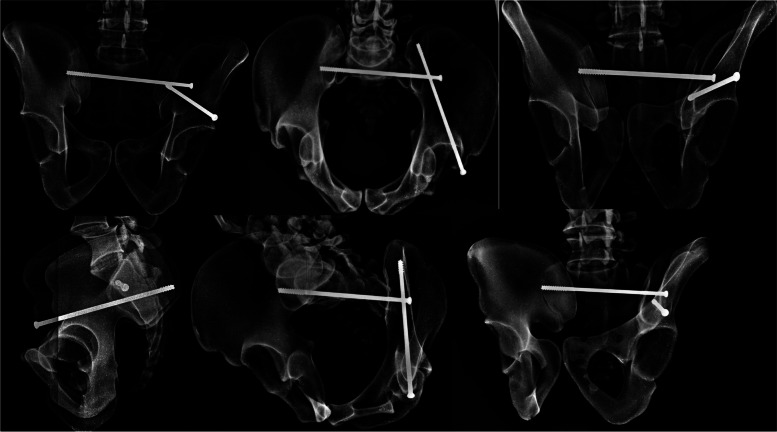
AP, Inlet, Outlet, Lateral, Obturator Inlet and Obturator Outlet X-Rays

An LC-II screw was first placed, followed by a trans-sacral screw into the first sacral segment. Repeat O-Arm spin demonstrated accurate placement of the screws. Each screw was contained completely within the sawbones model on the repeat spin. Visual inspection of the model confirmed no cases of cortical violation or screw malposition. Plain radiographs demonstrate screw containment within the bone model (Fig. 6) and positioned consistent with the pre-procedure plan.

## Discussion

It is important to note that this feasibility study serves only as an initial step to see if the MXSE robotic arm could be used to place screws into two commonly used osseus fixation pathways in a sawbones model. This study should not be interpreted in any way that the robot could or should be used in cases with actual patients in the ways described. Our study serves only to demonstrate the possible feasibility of the use of the robotic arm in off-label applications. Further cadaveric studies are needed with multiple screws placed in more than these two trajectories to further explore robotic arm assisted surgery for placement of screws in these and other osseus fixation pathways with rigorous measurements to evaluate accuracy of actual screw placement vs the planned trajectories. Other surgical techniques for the use of CT navigation for placement of similar screws typically rely on navigation of the 2.8- or 3.2-mm guidewire. The authors of the current study have observed that, especially as screw lengths increase, accurate navigation of the guidewire becomes unreliable and can lead to perforation of the model despite software indicating that the wire is contained within the bone. Maximizing the use of native instrumentation in the form of an opening burr followed by a navigated tap from the manufacturer may lead to more reliable screw placement in comparison with attempting to navigate guidewires, but further study is needed.

There are several potential advantages to a robotic arm surgical technique for placement of the screws. If data from pedicle screw placement can be extrapolated to the pelvis and acetabulum, it would not be unreasonable to expect that the use of the robotic arm would lead to accuracy as least as good as – if not better – than screws placed with fluoroscopy. Additionally, the use of robotic arm guidance may enable safe and accurate placement of implants in cases where fluoroscopic techniques prove inadequate due to obesity, overlying bowel gas or other factors that prevent adequate fluoroscopic imaging from being obtained.

## Conclusion

It is technically possible to place trans-sacral and LC-II style screws with the off-label use of the MXSE robotic arm. Further study is needed to clarify if other osseus fixation pathways are technically possible as well as characterize the accuracy of implant placement vs planned trajectories. In no way should this feasibility study imply that these techniques should be applied to actual patient care or used outside of an experimental, laboratory setting.

## Data Availability

Data sharing is not applicable ot this article as no datasets were generated or analysed during the current study.
